# Effects of Different Caffeine Dosages on Maximal Physical Performance and Potential Side Effects in Low-Consumer Female Athletes: Morning vs. Evening Administration

**DOI:** 10.3390/nu16142223

**Published:** 2024-07-11

**Authors:** Houda Bougrine, Achraf Ammar, Atef Salem, Khaled Trabelsi, Piotr Żmijewski, Haitham Jahrami, Hamdi Chtourou, Nizar Souissi

**Affiliations:** 1 High Institute of Sport and Physical Education Gafsa, University of Gafsa, Gafsa 2100, Tunisia; houdabougrine@live.fr; 2 Physical Activity Research Unit, Sport and Health (UR18JS01), National Observatory of Sports, Tunis 1003, Tunisiah_chtourou@yahoo.fr (H.C.); n_souissi@yahoo.fr (N.S.); 3 Department of Training and Movement Science, Institute of Sport Science, Johannes Gutenberg-University Mainz, 55099 Mainz, Germany; 4 High Institute of Sport and Physical Education Sfax, University of Sfax, Sfax 3000, Tunisia; trabelsikhaled@gmail.com; 5 Research Laboratory, Molecular Bases of Human Pathology, LR19ES13, Faculty of Medicine of Sfax, University of Sfax, Sfax 3029, Tunisia; 6 Research Laboratory, Education, Motricity, Sport and Health (EM2S), LR15JS01, High Institute of Sport and Physical Education of Sfax, University of Sfax, Sfax 3000, Tunisia; 7 Faculty of Physical Education, Gdansk University of Physical Education and Sport, 80-336 Gdansk, Poland; piotr.zmijewski@insp.pl; 8 Department of Psychiatry, College of Medicine and Medical Sciences, Arabian Gulf University, Manama 323, Bahrain; hjahrami@health.gov.bh; 9 Ministry of Health, Manama 410, Bahrain; 10 High Institute of Sport and Physical Education Ksar-Saïd, Manouba University, Mannouba 2010, Tunisia

**Keywords:** caffeine dose, diurnal variation, oral temperature, countermovement jump, agility, repeated sprint ability, rating of perceived exertion, handball, time of day, side effects

## Abstract

While previous studies have explored a range of factors governing the optimal use of caffeine (CAF) in athletes, limited research has explored how time of day (TOD) affects the ergogenic effects of various CAF dosages on physical performance. This study aimed to increase knowledge about how different recommended CAF doses (3 mg/kg vs. 6 mg/kg) ingested at different TODs affected maximal high-intensity physical performance and the perception of potential side effects in female athletes. In this double-blind, randomized, and counterbalanced study, 15 low CAF consumer athletes (aged 18.3 ± 0.5 y) underwent six trials, including three testing conditions assessed across two TODs: one in the morning (08:00 a.m.) and one in the evening (06:00 p.m.). During each condition, the participants ingested either a placebo, 3 mg/kg CAF (CAF (3 mg)), or 6 mg/kg CAF (CAF (6 mg)) capsules 60 min before each test with an in-between washout period of at least 72 h. In each trial, the participants performed a countermovement jumps test (CMJ), a modified agility t test (MATT), a repeated sprint ability (RSA), a rating of perceived exertion (RPE), and finally, a CAF side effects questionnaire. Our findings indicate the absence of an ergogenic effect on CMJ, MAT, and RSA performance in the evening after administering CAF (3 mg) or CAF (6 mg) compared to a placebo. Likewise, when CAF was ingested in the morning, there was an improvement in these performances with both CAF (3 mg) and CAF (6 mg), with greater improvement observed after CAF (6 mg). Additionally, neither the CAF dosage nor the TOD had a significant effect on the RPE. The occurrence of side effects increased significantly after the evening ingestion of CAF, particularly with a moderate dose of CAF (6 mg). Our findings indicate that the effectiveness of CAF depends on the TOD and CAF dosage. When ingested in the morning, a moderate dose of CAF (6 mg), rather than CAF (3 mg), is more effective in improving short-term physical performance without affecting CAF side effects in female athletes. Nevertheless, when ingested in the evening, neither dose was sufficient to enhance short-term physical performance, and both dosages increased the incidence of CAF side effects, particularly at a moderate dose.

## 1. Introduction

Caffeine (1,3,7-trimethylxanthine) (CAF) consumption in sporting contexts is currently widespread, regardless of the sport or athlete’s fitness level. Recent evidence indicates that three out of four elite athletes consumed CAF in all sport types before or during an athletic event [1,2,3]. The solid data showing the ergogenic impact of CAF in a variety of exercise scenarios are likely the reason for its widespread use in sports as a performance-enhancing stimulant for both aerobic and anaerobic performance [4]. The main hypotheses suggested to explain the advantageous effects of CAF on exercise situations that imply maximal strength production are two-fold. Firstly, the ergogenicity of CAF is associated with its ability to bind to adenosine receptors, which impedes the fatiguing effect of adenosine on the central nervous system [3]. Secondly, CAF enhances motor unit recruitment, further enhancing performance [4,5].

Recent data support the idea that the ingestion of this natural alkaloid as an ergogenic aid at a dose of 3–6 mg/kg improves performance in a broad range of short-term maximal exercise tasks in male and female athletes [3,5]. However, considerably less attention has been given to the importance of CAF’s effectiveness among female athletes and its relationship with anaerobic performance in ball games. Explorations into the effects of CAF on female team sport athletes have yielded valuable insights, yet they have also revealed gaps in the existing research. It has been shown that moderate doses of 5 or 6 mg/kg CAF were effective in enhancing short-term maximal performance among female athletes, including jumping, agility, and repeated sprint tests in female volleyball [6,7], handball [8,9], and football players [10]. While the consumption of 6 mg/kg of CAF did not significantly affect jumping performance [11,12] or agility [13], the same dose has been shown to enhance jumping [8,10,14], agility [8], and repeated sprinting [10] performances among female athletes. It has been revealed recently that a moderate dose (5 mg/kg of CAF) can improve jump performance in female volleyball players [7]. Other studies revealed the effectiveness of a lower dose, equivalent to 3 mg/kg or less, in these performances in basketball and rugby [15]. Similarly, Pfeifer et al. [16] and Stojanovic et al. [17] did not reveal any significant effects of lower CAF supplementation, while Ali et al. [11] and Lara et al. [10] indicated that even a moderate dose did not enhance maximal performance among female team ball athletes. The administration of low doses has been revealed to have minimal to no effect on repeated bouts [5,14,18], agility [5,13,14], or jumping [16,17]. Conversely, the same dosage has been reported to enhance jumping [5,6] and agility [6] performances among female team ball athletes. These discrepancies could result from variations in the doses (low, moderate, or higher) and mode of CAF administration (CAF forms, timing of ingestion, time of day, washout duration, administration protocol, CAF restriction duration, blinding of CAF ingestion, and CAF doses). Moreover, the diversity of participant characteristics (physical levels, sample size, sex, discipline, age, habitual consumption) under investigation could in part explain this divergence.

To strengthen our understanding of whether moderate or lower dosages should be administered to female athletes to enhance their short-term physical performance, further data comparing two or more doses within the same participant group are needed. Ultimately, the comparison of the ergogenic effects of low and high CAF doses is complicated by the variability in performance enhancement arising from varied CAF dosages among diverse studies and individuals. As a result, additional studies are needed to determine how various CAF dosages affect the same individuals to draw more precise comparisons and reliable findings. In this context, a similar effectiveness of CAF intake in improving lower body muscular endurance [19] and average peak power scores during repeated sprint tests on a cycle ergometer was revealed after the ingestion of both 3 and 6 mg/kg [20] among trained female team sports players. With the same CAF doses, a recent study indicated the superiority of 6 mg/kg compared to 3 mg/kg of CAF in enhancing different aspects of short-term high-intensity exercise, such as jumping, agility, and repeated sprint bouts [6]. However, a moderate (5 mg/kg) dose of CAF was greater than 2 mg/kg in decreasing pain perception during muscular endurance tests among female karate athletes [21]. Despite these findings, the use of a single dose raises doubts about whether higher doses may yield more pronounced effects on performance. Furthermore, the variations in outcomes associated with various doses of CAF on critical aspects of high-intensity exercise performance in team sports remain insufficiently documented, presenting a promising avenue for further investigation. Although previous studies have examined a range of factors that could contribute to the optimal use of this stimulant, such as doses and protocols of administration, it is interesting to note that while studies have indicated that athletes’ CAF effectiveness fluctuates with the time of day (TOD) [8,22], there is surprisingly little information available in this field, particularly regarding female athletes and various CAF dosages [23]. Notably, there are conflicting results about how TOD affects the effectiveness of CAF in enhancing athletic performance. For example, a small dose of 3 mg/kg of CAF has been demonstrated to reverse morning neuromuscular declines, boosting performance to levels comparable with those in afternoon trials [24]. However, it has been recently observed that the same dose of CAF induced the use of more fat as fuel during exercise for trained individuals, regardless of the TOD [25]. Additionally, previous studies have suggested that moderate doses of 5 mg/kg [26] or 6 mg/kg [22,27,28] may be more effective in the morning than in the afternoon. Lopes-Silva et al. [29], however, showed that supplementing with 5 mg/kg of CAF did not increase performance in the afternoon or prevent a decline in performance on the repeated sprint test in the morning. This is particularly evident in female athletes, where 6 mg/kg of CAF has been shown to enhance performance more effectively in the morning than in the afternoon, especially among female handball players [8,9]. Remarkably, to the best of our knowledge, no investigation has examined the effects of different doses at various times of day on athletes’ short-term, high-intensity physical performance. This variable is essential for optimizing the beneficial impacts of this stimulant and reducing any possible negative effects. Therefore, this substance should be considered when it is administered throughout two games on consecutive days or for afternoon training sessions. Although consumed, it can cause adverse side effects such as anxiety, nausea, headaches, or insomnia [30]. Nevertheless, there has not been sufficient discussion of the potential negative effects of consuming CAF, especially at the dosages used to improve performance [31,32], and of the TOD of CAF administration among female athletes. Consequently, the purpose of this study was to investigate how different CAF doses (3 mg/kg vs. 6 mg/kg) at different times of the day (morning vs. evening) affected the maximal high-intensity physical performance and perceived exertion and potential side of female athletes. Based on the available literature, our hypothesis is that (i) the consumption of both 3 and 6 mg/kg of CAF may enhance the short-term high-intensity performance of female athletes, particularly in the morning but not in the evening; (ii) the pre-exercise intake of 6 mg/kg of CAF could be more synergistic than 3 mg/kg in both the morning and evening in low CAF consumers’ female athletes; and (iii) the evening pre-exercise intake of 6 mg/kg of CAF could increase the occurrence of CAF side effects.

## 2. Materials and Methods

### 2.1. Participants

The G*Power software (version 3.1.9.6; Kiel University, Kiel, Germany) [33] was used to predetermine the necessary sample size, following the recommended guidelines put forward by Beck [34]. With a desired statistical power (β) of 0.95, the significance level (α) was set at 0.05. Based on Bougrine et al. [8] and Karaygit et al. [20], the effect size was estimated to be 0.5. It was determined that a sample size of at least 12 athletes would be sufficient to achieve the necessary statistical power, reducing the probability of a type 2 statistical error.

The sample consisted of 20 female participants after screening 44 surveys. This number was reduced to 15 because 5 participants dropped out due to menstrual cycles (3 athletes) and personal reasons (2 athletes). The study included young, well-trained female handball players (aged 18.3 ± 0.5 y [18–19 y]; height: 1.65 ± 0.6 m; weight: 60.2 ± 5.7 kg; BMI: 22 ± 2.1 kg/m^2^) who volunteered to participate and met the inclusion criteria. Only female athletes aged 18–30 years who were fully able to exercise and attend all trials were included. Only athletes with lower CAF consumption and daily habitual intake of caffeinated beverages (e.g., coffee, espresso) less than 0.99 mg/kg/day (low consumers), but higher than 25 mg/day (not naïve consumers) were included [5,35,36,37]. All athletes had engaged in handball for a minimum of 3 years (5.5 ± 0.7 y), with at least 3 sessions per week in the last 6 months (4.4 ± 0.5 sessions/week) and maintained regular menstrual cycles over the last 6 months (27.8 ± 2 days). Furthermore, athletes were excluded if they (a) used oral contraceptives, medications such as pills, patches, injections, implants, or intrauterine devices in the previous three months because the metabolism of CAF interacts with the use of oral contraceptives by extending the duration of its effects [38]; (b) had a positive alcohol and/or smoking status and followed strict dietary regimens in the previous three months that could affect hormone balances or physical performance; (c) had a history of diseases, used stimulants, narcotics, mind-altering drugs, or nutritional enhancers (e.g., creatine, nitrates, or bicarbonate); or (f) were allergic to CAF. To ensure group homogeneity and prevent circadian typology and sleep issues from influencing study outcomes, all participants met the following inclusion criteria: (i) only those identified as having a “neither type” chronotype (47.5 ± 5.5 scores) were selected for our study using the self-assessment questionnaire developed by Horne and Ostberg [39]; (ii) only participants with a normal average sleep duration (7.4 ± 0.5 h) and sleep quality (scores all below 5) according to the Pittsburgh Sleep Quality Index (PSQI) questionnaire in the month leading up to the experimental procedure [40]; (iii) only participants with lower CAF consumption (0.78 ± 0.2 mg/kg/day) according to a reliable semi quantitative self-reported CAF intake questionnaire in the four weeks leading up to the initiation of our experiment; and (iv) each athlete was evaluated during the follicular phase, the luteal phase, or both phases of their menstrual cycle using the mobile application Mycalendar^®^ (Period Tracker, version (1.75.305.GP), Simple Design Ltd., Wong Chuk Hang, Hong Kong) mobile application, which records significant events during the menstrual cycle [41].

All the athletes were informed about all the details of the experiment, including the schedules, supplementation, and tests that they would have to participate in advance. The University of Jendouba’s local research ethics committee (054-2023) approved all protocols and methods that followed the most recent edition of the Declaration of Helsinki and the ethical and procedural requirements for human chronobiology research [42].

### 2.2. Experimental Design

**Figure 1:**
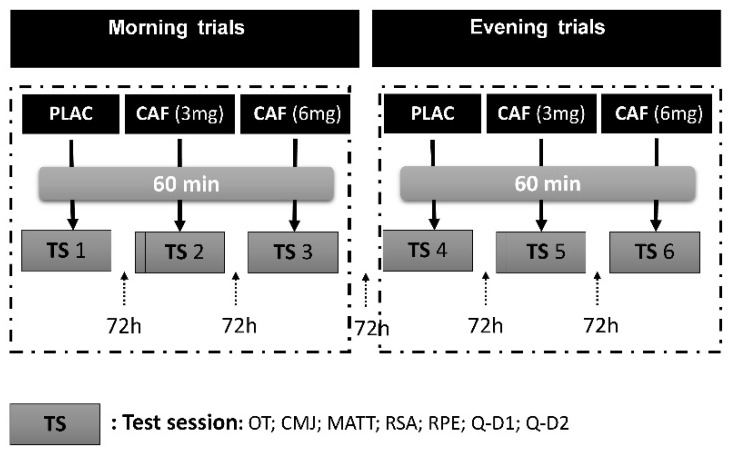
Study design: PLAC: placebo; CAF: caffeine; CAF (3 mg): 3 mg/kg of CAF; CAF (6 mg): 6 mg/kg of CAF; TS: test session; OT: oral temperature; CMJ: countermovement jump test; MATT: modified agility t test; RSA: repeated sprint ability test; RPE: rating of perceived exertion; Q: questionnaire of CAF adverse side effects; Q-D1: questionnaire on the same day; Q-D2: questionnaire on the following day.

In a double-blind, placebo-controlled, randomized design, the experimental protocol of our study consisted of six experimental sessions conducted across three testing conditions. During each condition, the subjects ingested one of the following: a placebo (PLAC), 3 mg/kg of CAF (CAF (3 mg)), or 6 mg/kg of CAF (CAF (6 mg)) one hour before each test in two different sessions: one in the morning (a.m.) (from 08:00 a.m. to 09:00 a.m.) and one in the evening (p.m.) (from 06:00 p.m. to 07:00 p.m.). Throughout the six experimental sessions, a minimum washout period of 72 h between each session was allowed to ensure full recovery and elimination of substances (Figure 1). Both supplements were unflavored, uncolored, and odorless. These specific times were selected to coincide with the batyphase and acrophase, representing physical performance and oral temperature for this chronotype, respectively [8,43]. These specific doses were selected based on previous studies reporting the effectiveness of these doses on female athletes [5,8,14]. Using a free online software resource (www.randomization.com) (accessed 10 September 2023), initially the conditions (CAF dosage or placebo administration), followed by the order of the time of day (TOD) for the testing trials were randomized. All participants underwent testing under all conditions of different Condition/TOD combinations. A study team member who was not directly involved in data collection carried out the blinding and randomization of the trial sessions, thus both participants and researchers were blinded to the trials once assigned to interventions.

Athletes underwent familiarization and data assessment visits two weeks before the experiment. Leading up to the study, they were familiarized with the experimental protocol to minimize learning effects and ensure high-quality results. Before the trials, the athletes’ weight and height were assessed using a Tanita BC-545n digital scale (Tanita Corporation, Arlington Heights, IL, USA) to the nearest 0.1 kg. Body weight was assessed early in the morning with minimal clothing, no shoes, and an empty stomach after defecation and urination before the weighing process to ensure the accuracy of the body mass measurements [44]. The mean body weight measured in two familiarization sessions before the experimental trials was used for the dosage calculations throughout the experiment.

Each test session began just 60 min after the capsules were ingested. This duration corresponds to the peak plasma CAF concentration, which usually occurs between 15 and 120 min following oral consumption [45]. Caffeine is rapidly absorbed into the bloodstream via the gut, generally reaching peak plasma levels 60 min after administration [3]. During each session of the six testing trials, the athletes began the experimental procedure by having a digital clinical thermometer (Omron, Paris, France; accuracy ±0.05 °C) inserted sublingually for at least 3 min to assess their resting oral temperature. Following this, participants underwent a standardized warm-up (~10 min including rest). The warm-up included 3 min of jogging (at 8–10 km/h). Jogging was followed by 3 min of whole-body dynamic stretching, 2 min of sprinting (inkling, high-knee, back-kicking, and skipping), and 2 min of sprinting. Participants were then tested during each session in the same order: countermovement jumps test (CMJ), modified agility t-test (MATT), repeated sprint ability (RSA), rating of perceived exertion (RPE), and finally, the CAF negative effects questionnaire. A 5 min rest period was provided between these tests to ensure adequate recovery. After completion of the test battery, the athletes were asked to estimate which supplement (PLAC or CAF (3 mg) or CAF (6 mg)) they had been given at the beginning of the test trial.

During each experimental trial, the athletes received either placebo capsules containing an inert substance (Cellulose; Guinama 6, Valencia, Spain) or an appropriate dose of CAF capsules (Bulk Powders, Colchester, UK). The capsules were ingested with a cup of water (100 mL) one hour before the trial and at least one hour after the last meal to ensure consistent absorption, aligning with the pharmacokinetics of CAF [45]. To minimize potential gastrointestinal discomfort that could be caused by the fasting state (at least 6 h of fasting for both trials), participants were provided with a standardized breakfast in the morning trials or a snack during the afternoon trials which was consumed 120 min before each trial. Taking into account their usual pretrial nutrition routine, with a daily caloric intake of approximately 2093.8 ± 347.72 Kcal, this breakfast meal or snack amounted to approximately 500 Kcal. Furthermore, players were asked to maintain a regular food diary for 48 h before each test, including the testing day. Before the first trial, they collected a 24 h record of their routines and dietary consumption. After that, photocopies of the records were prepared and distributed to each athlete to guarantee dietary consistency in subsequent sessions. A list of food and drink items containing CAF was given to all athletes to avoid contamination during the 24 h and the day of testing. Before the start of each session, the participants verbally agreed to follow the prescribed dietary instructions. They were instructed to maintain their regular training schedule, refrain from performing strenuous exercise the day before each trial, and abstain from stimulants and CAF for the full 24 h leading up to and including the testing day. In addition, participants were instructed to maintain their regular lifestyle hydration and sleep habits for at least 7 h the night before each experiment. To ensure consistency in the measurements, each assessment was carried out at the same TOD on an identical indoor court with the same testing equipment under the supervision of the same investigators and according to the same protocol. All six of our experimental sessions were conducted in an indoor court with consistent ambient temperatures (approximately 28 °C) and relative humidity (52%).

### 2.3. CMJ

As described by Bougrine et al. [5], the athletes were asked to perform a quick vertical jump involving a downward preparatory movement and an upward propulsive phase from an upright posture. This test was performed using Optojump-next equipment (Bolzano, Italy) and the accompanying Microgate software (Optojump software, version 1.10.50). The height of the jump, measured in centimeters, was the single piece of information used in the investigation. All participants completed three trials, with a two-minute break in between. For analysis, the highest jump recorded during these three attempts was retained.

### 2.4. MATT

A timed gate (Witty, Microgate^®^, Bolzano, Italy) was used for conducting the modified agility t-test, which included multidirectional sprinting, shuffling, and backpedaling [46]. Starting with a 5 m linear sprint to cone B, the athletes had to shuffle 2.5 m left to cone C, 5 m right to cone D, shuffle 2.5 m left to cone B again, and then shuffle 5 m linearly backpedal to cone A. A-B was 5 m long, and the distance between B-C and B-D was 2.5 m. The total distance traveled for each trial was 20 m. The fastest time from two trials, separated by three minutes of rest, determined the MAT performance in our analysis.

### 2.5. RSA

As described by Bougrine et al. [5], a repeated sprint ability (RSA) test involving six maximal 2 × 12.5 m shuttle sprints, each separated by 20 s of passive rest, was conducted. The timing gates (Witty, Microgate^®^, Italy) recorded the timings. The best sprint (RSA peak) and the mean time of six sprints (RSA mean) were retained for analysis.

### 2.6. RPE

A Borg scale [47] of ten points was used in this study. The French version of the scale states that a score of zero denotes rest, equivalent to sitting in a chair, and a score of ten denotes extremely intensive physical activity [48]. The RPE was verbally evaluated following each of the RSA test’s 25 m shuttle sprints. The final score was then determined by calculating the mean of these six RPE measurements.

### 2.7. CAF Side Effects Questionnaire

Following the test session (Q-Day 1), players were instructed to complete a nine-item questionnaire employing a dichotomous (yes/no) response scale concerning their perceptions of potential adverse effects associated with pre-exercise CAF intake [49]. Furthermore, the following day (Q-Day 2), the athletes completed the same questionnaire again, focusing on the side effects linked to caffeine intake during the hours following the trial.

### 2.8. Habitual CAF Intake Assessment

As recently suggested by Filip et al. [35], habitual CAF intake was assessed using the following criteria: (i) using the validated Food Frequency Questionnaire (FFQ), based on household quantities over four weeks before the experiment [50]; (ii) calculating daily CAF intake for each athlete, related to their body mass, by a nutritionist using nutritional tables; and (iii) only participants deemed low consumers with daily CAF consumption between 25 mg/day and 0.99 mg/kg/day were included in our data analysis.

### 2.9. Statistical Analysis

GraphPad Prism 8 (GraphPad Software, San Diego, CA, USA) was used to generate the figures. STATISTICA software version 12 (StatSoft, OK, USA) was used to evaluate the data that were collected in this investigation. The means ± standard deviations (SDs) were calculated for each variable. A normal distribution of all the data was verified by the Shapiro–Wilk test. To examine the impact of CAF on physical performance, a two-way ANOVA [3 (Dosage) × 2 (TOD)] with repeated measures was performed. Where applicable, Tukey’s HSD post hoc test was used to assess for significant differences between conditions in each time of day, and between time of days in each condition. The effect size statistic (ηp^2^) was used to determine the magnitude of the difference between age groups. The effect sizes were classified using the following criteria: 0.01 denoted a small effect size, 0.06 represented a moderate effect size, and 0.14 indicated a large effect size [51]. Standardized effect size (Cohen’s d) analysis was used to interpret the magnitude of differences between variables and classified them according to Hopkins [52] as trivial (d ≤ 0.20), small (0.20 < d ≤ 0.60), moderate (0.60 < d ≤ 1.20), large (1.20 < d ≤ 2.0), very large (2.0 < d ≤ 4.0), and extremely large (d > 4.0). The significance level was considered to be *p* ≤ 0.05.

## 3. Results

### 3.1. Oral Temperature (OT)

**Figure 2:**
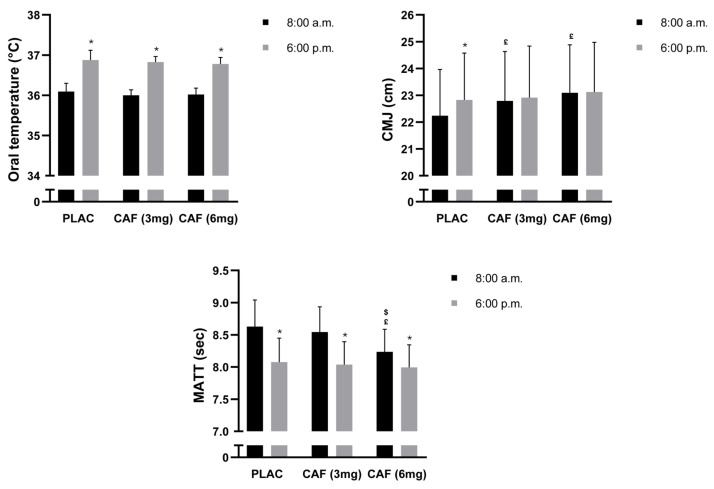
The mean ± SD values of the oral temperature, countermovement jump test (CMJ), and modified agility t-test (MATT) were recorded under three conditions: PLAC: placebo, CAF (3 mg): 3 mg/kg of CAF, and CAF (6 mg): 6 mg/kg of CAF at 8:00 a.m. and 6:00 p.m. * (*p* < 0.001): significant difference compared to 08:00 a.m. (under the same conditions). **^£^** (*p* < 0.001): significant difference compared to PLAC (at the same time of day). **^$^** (*p* < 0.001): significant difference compared to CAF (3 mg) (at the same time of day).

Statistical analysis of OT revealed a moderate significant main effect of TOD (F (1, 14) = 398; *p* < 0.001; ηp^2^ = 0.96). However, there were no significant effects of dosage (F (2, 28) = 3; *p* = 0.06; ηp^2^ = 0.17) or the dose × TOD interaction (F (2, 28) = 1; *p* = 0.40; ηp^2^ = 0.07) on OT. Further analysis indicated that evening OT was greater than morning OT across the different dosage conditions (all *p* < 0.001) (Figure 2).

### 3.2. CMJ

Statistical analysis indicated a small significant main effect of dosage (F (2, 28) = 12.12; *p* < 0.001; ηp^2^ = 0.46), TOD (F (1, 14) = 21.25; *p* < 0.001; ηp^2^ = 0.60), and the dosage × TOD interaction (F (2, 28) = 7.44; *p* < 0.01; ηp^2^ = 0.34) on CMJs. Post hoc analysis revealed that evening CMJ was greater than morning CMJ during the PLAC condition (2.6%, *p* < 0.001). Compared to the morning PLAC condition, the CMJ performance was better after the ingestion of CAF (3 mg) (2.5%, *p* < 0.001) and CAF (6 mg) (3.8%, *p* < 0.001). However, no difference was detected after evening ingestion of CAF (3 mg) (*p* >0.05) or CAF (6 mg) (*p* > 0.05) compared to that of PLAC at the same TOD (Figure 2).

### 3.3. MATT

A two-way ANOVA demonstrated a moderate significant main effect of dosage (F (2, 28) = 45.30; *p* < 0.001; ηp^2^ = 0.76), TOD (F (1, 14) = 51.48; *p* < 0.001; ηp^2^ = 0.78), and the dosage × TOD interaction (F (2, 28) = 31.71; *p* < 0.001; ηp^2^ = 0.69) on MATT, indicating that CAF dosage interferes with the effect of TOD on agility performance. Evening MATT performance was better than morning MATT performance under the PLAC condition (−6.4%, *p* < 0.001). MATT performance was improved after the morning consumption of CAF (6 mg) (−4.5%, *p* < 0.001) compared to the placebo. However, CAF (3 mg) in the morning did not lead to a significant increase in MATT performance (*p* > 0.05). Furthermore, no significant differences in MATT performance were revealed after evening ingestion of either CAF (3 mg) (*p* > 0.05) or CAF (6 mg) (*p* > 0.05) compared to that of the PLAC (Figure 2).

### 3.4. RSA Mean

**Figure 3:**
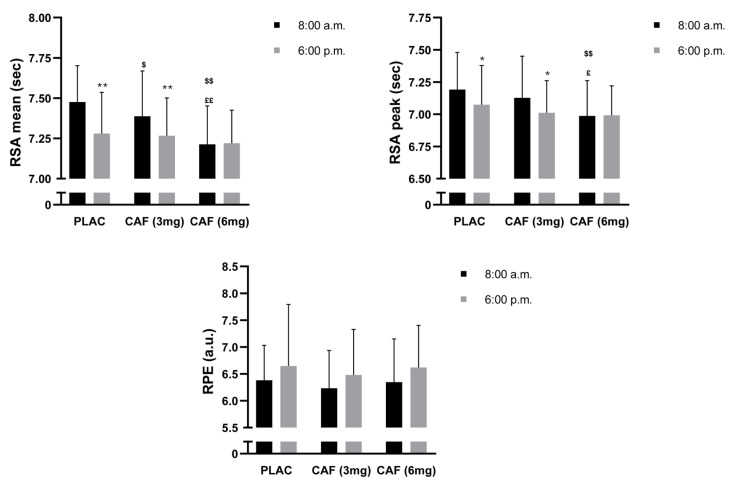
Mean ± SD values of repeated sprint ability (RSA) performance and rating of perceived exertion (RPE) scores measured under three conditions: PLAC: placebo, CAF (3 mg): 3 mg/kg of CAF, and CAF (6 mg): 6 mg/kg of CAF at 8:00 a.m. and 6:00 p.m. * (*p* < 0.05); ** (*p* < 0.001): significant difference compared to 08:00 a.m. (in the same conditions). **^£^** (*p* < 0.01); **^££^** (*p* < 0.001): significant difference compared to PLAC (at the same time of day). **^$^** (*p* < 0.05); **^$$^** (*p* < 0.001): significant difference compared to CAF (3 mg) (at the same time of day).

The ANOVA revealed a small significant main effect of dosage (F (2, 28) = 17.64; *p* < 0.001; ηp^2^ = 0.55) and TOD (F (1, 14) = 8.80; *p* < 0.05; ηp^2^ = 0.38) on the RSA mean. Moreover, there was a significant main effect of the dosage × TOD interaction (F (2, 28) = 15.60; *p* < 0.001; ηp^2^ = 0.52) on the RSA mean. There was a significant difference between morning and evening RSA mean performance under the PLAC condition, with better performance observed during the evening (−2.6%, *p* < 0.001). The post hoc test demonstrated that compared with those of PLC, the dosages of CAF (3 mg) (−1.1%, *p* < 0.05) and CAF (6 mg) (−3.5%, *p* < 0.001) improved the mean RSA. Evening administration of CAF (3 mg) or 9-CAF did not improve the mean RSA compared to that in the PLAC group (both *p* >0.05) (Figure 3).

### 3.5. RSA Peak

Statistical analysis of the RSA peak did not reveal a significant main effect of TOD (F (1, 14) = 1.19; *p* = 0.29; ηp^2^ = 0.07). However, a small significant main effect of dosage (F (2, 28) = 7.83; *p* < 0.01; ηp^2^ = 0.35) and a trivial significant main effect of the dose × TOD interaction (F (2, 28) = 3.52; *p* < 0.05; ηp^2^ = 0.20) on the RSA peak were observed. Post hoc analysis revealed that the evening RSA peak was greater than the morning RSA peak during the PLAC condition (−1.6%, *p* < 0.05). Compared to the morning PLAC condition, the RSA peak was greater after the consumption of CAF (6 mg) (−2.8%, *p* < 0.001). However, no difference was detected after evening ingestion of CAF (3 mg) (*p* > 0.05) or CAF (6 mg) (*p* > 0.05) compared to that of PLAC at the same TOD (Figure 3).

### 3.6. RPE

There were no significant main effects of dosage (F (2, 28) = 0.54; *p* = 0.58; ηp^2^ = 0.03) or TOD (F (1, 14) = 1.76; *p* = 0.20; ηp^2^ = 0.11) on the RPE score. Additionally, there was no significant main effect of the dosage × TOD interaction (F (2, 28) = 0.005; *p* = 0.99; ηp^2^ = 0.0003) (Figure 3).

### 3.7. CAF Side Effects Questionnaire

There was a low occurrence of CAF side effects in both morning and evening trials, ranging from none to 13.33%, following the PLAC trials (Q-D1) and on the next day (Q-D2). Minor side effects, ranging from none to 13.33%, were reported after the administration of CAF (3 mg) at Q-D1 in both the morning and evening trials. On the following day, while a minor occurrence of CAF (3 mg) side effects (0–13.33%) was reported in the morning, a greater occurrence was observed when CAF was ingested in the evening (6.66–40%). When ingested in the morning, the CAF (6 mg) dose resulted in a similar occurrence of adverse symptoms on both the same day and the following day, ranging between 0 and 20%. However, when the same dosage was ingested in the evening, a greater prevalence of side effects was observed during the same day (6.66–33.33%) and the following day (13.33–46.66%). On the same-day test, there were increased reports of heightened tachycardia (33.33%), headaches (20%), and gastrointestinal problems (26.66%). Similarly, gastrointestinal problems (26.66%), headaches (40%), and insomnia (46.66%) increased on the day after afternoon CAF (6 mg) ingestion (Table 1).

Notably, athletes did not correctly identify the dosage of pre-exercise CAF intake, with correct identification ranging from 20% for CAF (3 mg) trials to 13.33% for CAF (6 mg) trials. Surprisingly, 26.66% of players correctly identified that they had taken a placebo. However, none of the athletes were able to correctly identify all six trials in which they took part, suggesting that the study’s blinding was preserved (Table 1).

## 4. Discussion

The current study represents a pioneering effort to analyze the effect of the TOD and various doses of CAF intake on short-term maximal performance and side effects among low CAF consumer female athletes. The main findings of the current study revealed that (i) a significant moderate improvement in agility and RSA performance was evident with the 6 mg/kg dosage, whereas a trivial improvement was observed with the 3 mg/kg dosage in the morning; (ii) both CAF doses significantly improved CMJ performance without affecting the perceived exertion scores (RPE) or oral temperature; and (iii) evening CAF ingestion with both doses did not seem to enhance these performances but was associated with a greater occurrence of side effects, particularly after moderate dose intake among individuals with low CAF consumption habits. The aforementioned findings may have implications for female athletes seeking to enhance their short-term maximal performance without experiencing any negative side effects from pre-exercise CAF intake.

### 4.1. TOD Effects

Our findings revealed that short-term maximal performance is TOD-dependent, with evening performance superior to morning performance, which is in line with the findings of Ayala et al. [53], indicating that 04:30–06:30 p.m. is the most appropriate TOD for several aspects of physical activity. While some studies [54,55] did not report any daily fluctuations in physical maximal exercise among female athletes, several studies have indicated diurnal variations in these parameters among female athletes [8,9,56,57]. Although the precise mechanism of evening effectiveness is not fully defined, the most accepted hypothesis proposes that variables related to body temperature [58,59] and physiological, psychological, and metabolic rhythms [60,61] peak in the afternoon. It is important to note that different participant chronotypes, having distinct internal biological clocks and motivations from the moment they woke up [62] and regular training times [43], were reported to have a direct effect on this daily fluctuation.

### 4.2. CAF Dosage Effects

Our results indicated that both CAF doses significantly improved morning CMJ performance compared to a placebo, which aligns with our hypothesis. This outcome is consistent with previous data indicating that 3 mg/kg [6,14] and 6 mg/kg [5,10] CAF were effective in enhancing this performance among female athletes. Although Ali et al. [11] and Fernández-Campos et al. [12] reported that 6 mg/kg was not effective in enhancing CMJ among female athletes, Bougrine et al. [5] revealed that both 3 and 6 mg/kg were sufficient to improve CMJ in a sample of female team ball athletes. Recent findings indicated that 5 mg/kg CAF intake has been linked to improved CMJ performance among female volleyball players [7].

Regarding agility and repeated sprint ability, the moderate dose of 6 mg/kg of CAF seems to be more effective than 3 mg/kg in enhancing this performance, which aligns with previous findings in female team ball sports indicating the superiority of a 6 mg/kg dose over 3 mg/kg on maximal performance [5]. Contrary to our hypothesis, this result was similar to the results of a recent meta-analysis that established the efficacy of a CAF intake ranging from 3 to 6 mg/kg in female team sports [63]. Although a lower dose of CAF did not enhance agility [16,17,64] or repeated sprints [15,16,17] in female athletes, interestingly, the same dosage was shown to have beneficial effects on agility [6]. Furthermore, a moderate dose of 6 mg/kg did not seem to be effective for agility [13,14] or repeated sprint ability [14,18] among female athletes. However, contradictory effects were reported in other studies in which the same dose was found to improve agility [8] and repeated sprinting [10]. Notably, it is hypothesized that the effects of different CAF doses could be intricately linked to the nature of the physical task and its duration. Lower doses seem to be sufficient to enhance performance in simple physical tasks such as jumping. Nevertheless, its effectiveness in more complex tasks, such as agility or repeated sprint ability, requires a higher dose of CAF. Furthermore, while low CAF dose intake (≤3 mg/kg) mainly enhances performance due to its direct modulation of the central nervous system [65], higher CAF dosage effectiveness could be explained by increasing calcium release from the sarcoplasmic reticulum [66]. Recent research has shown that both low (3 mg/kg) and moderate (6 mg/kg) doses of CAF can improve lower-body muscular endurance [19]. Additionally, Arazi et al. [21] reported that a higher dose of CAF (5 mg/kg) was more effective at reducing pain perception during muscular endurance tests in female karate athletes than a lower dose of 2 mg/kg. Bougrine et al. [5] demonstrated that a pre-exercise CAF intake of 6 mg/kg improved short-term maximal performance in female athletes, while Karayigit et al. [20] reported that both low and moderate doses of CAF increased average peak power during repeated sprint tests in trained female team sports players.

### 4.3. CAF and TOD Effects

Regarding CAF effectiveness across different times of day, our findings indicated that both doses were effective in the morning, with a better enhancement for 6 mg/kg CAF intake. Contrary to our hypothesis, which suggested that a pre-exercise intake of 6 mg/kg of caffeine (CAF) might be more synergistic than 3 mg/kg in both the morning and evening, our findings indicated that neither dose was effective after evening intake. Notably, while previous data suggest that the effectiveness of CAF for athletes depends on the TOD [8,22], there is surprisingly limited information on this topic, especially concerning female athletes and different CAF doses. Interestingly, there are conflicting findings regarding how TOD influences the ability of different doses of CAF to enhance athletic performance. Although Mora-Rodríguez et al. [24] revealed that a small dose of 3 mg/kg of CAF has been shown to counteract morning neuromuscular performance declines to levels comparable to those in afternoon trials, Muñoz et al. [25] indicated that the same dose of CAF induces increased fat utilization during exercise for trained individuals, regardless of the TOD. Souissi et al. [26] demonstrated the effectiveness of a moderate dose of 5 mg/kg in the morning rather than in the afternoon; however, Lopes-Silva et al. [29] showed that supplementation with the same dose (5 mg/kg) did not increase performance in the afternoon or prevent a decline in performance on the repeated sprint test in the morning. Previous investigations have suggested that moderate doses of 6 mg/kg of CAF might be more effective in the morning than in the afternoon [22,27,28]. This effect was evident in female athletes, where 6 mg/kg of CAF was found to enhance performance more effectively in the morning than in the afternoon, especially among female handball players [8,9].

While the decline in morning performance, a widespread issue in sports, has attracted attention and emerged as a research topic, CAF intake has emerged as an effective strategy for mitigating this decline in maximal physical performance [8,9,22]. It is hypothesized that CAF’s effectiveness peaks when performance is at its lowest values, which is often the case in the morning for the present study’s participants’ chronotypes (neither chronotype). This hypothesis was further confirmed by observations of the efficacy of CAF in enhancing physical performance, yielding results similar to those observed in the afternoon [8,22] or following a normal night of sleep, even in instances of partial sleep deprivation [67]. Furthermore, similar results were noted when CAF was consumed directly after the month of Ramadan, suggesting its potential efficacy in restoring performance levels post-fasting [9]. Notably, studies indicate that the effects of CAF on an individual’s circadian rhythm, or chronotype, may vary depending on how an individual responds to exercise [37]. It is possible that the “morning chronotype” will be more CAF ergogenic than the “night chronotype,” or vice versa [68]. The presence of CAF in the morning may mimic the physiological conditions of the evening for evening chronotypes, thereby improving performance [69]. Nevertheless, no research has focused on this relationship. Investigating the effects of CAF on participants’ chronotype during exercise, particularly concerning varying dosages among female athletes, may help develop tailored CAF consumption strategies depending on the TOD.

Moreover, melatonin is a hormone that regulates circadian rhythms and is influenced by CAF. Melatonin regulates sleep and the biological clock; it is primarily secreted at night. It has been revealed that CAF can inhibit the pineal gland’s ability to produce melatonin. As a result, early gene expression in the central circadian pacemaker in the SNC is triggered, and melatonin levels are decreased [18]. These mechanisms highlight the role of CAF in melatonin-mediated circadian rhythm regulation. However, further research is needed to determine its specific effect on the decline in morning performance [23,70]. On the other hand, the hypothesis that CAF directly influences circadian rhythms in the absence or isolation of ambient light exposure is not supported by enough evidence. The CAF probably affects the circadian rhythm of the body indirectly by making it more sensitive to phase shifts caused by light [71]. According to Hilaire and Lockley [72], however, there is little evidence to suggest that the CAF can independently change the amplitude or timing of the circadian clock when there is no light. While recent data on animals in continual darkness did not show any variations in the activity patterns of those given CAF and those who did not [73], it is logistically difficult to investigate the circadian effects of CAF in the absence of light in human investigations. Furthermore, it is plausible that the benefits of CAF observed in humans even in the presence of light involve improvements in the circadian response to light [23].

### 4.4. CAF Side Effects

Our findings are in line with our hypothesis and previous studies indicating that lower dosages were related to fewer negative effects, whereas higher dosages were associated with an increased incidence of adverse outcomes in both the morning [5,8] and afternoon [14,37,74]. Previous evidence indicated that higher dosages of CAF taken in the morning may lead to insomnia [49]. Similarly, it has been reported that while moderate to low dosages of CAF (6 mg/kg) are consumed, the incidence of adverse effects, mainly insomnia and tachycardia/heart palpitations, may increase as much as 34% according to a recent meta-analysis that investigated the effects of CAF supplementation in athletes [75].

The high incidence of negative side effects in the current study, mainly related to moderate doses, may be attributed to habitual CAF use. Frequent CAF users may develop tolerance to some of the substance’s physiological effects [76], indicating that regular CAF intake among athletes might have a reduced impact on sleep parameters and side effects. Thus, it can be speculated that low-CAF consumers in our study could be more sensitive to CAF intake than habituated athletes, which could explain in part the high occurrence of side effects. 

A recent study among female athletes revealed a positive correlation between morning CAF intake and the occurrence of side effects, including gastrointestinal problems and sleep troubles. Higher occurrences were noted after the intake of 9 mg/kg than after the intake of 6 or 3 mg/kg, with 6 mg/kg also showing a greater occurrence than 3 mg/kg [5]. It has been shown that while a lower dose produces positive emotions, a higher dose heightens feelings of tension, irritability, anxiety, and nervousness [77]. It is intriguing to note that a recent study established a direct link between higher levels of CAF intake and increased anxiety symptoms in women, albeit not in men [78], which could explain in part the greater prevalence of these side effects in our participants.

Notably, it is hypothesized that the withdrawal period of more than 24 h in our study could explain in part the occurrence of these side effects in morning sessions regardless of habitual CAF intake. Withdrawal symptoms from CAF usually manifest 12–48 h after the last intake and result in different clinical outcomes [79]. When regular CAF consumers limit their intake, the nocebo effect may play a role in the frequency of adverse side effects that are reported [80]. It has been reported that studies including brief durations of CAF deprivation of less than 24 h may find that participants’ initial states are affected, as the half-life of CAF might vary from 3 to 7 h [81]. Because it is close to participants’ bedtime, this half-life of evening-ingested CAF may be the primary reason for the incidence of additional side effects in the evening, particularly insomnia, which may be mainly explained by stimulating noradrenaline and adrenaline in the adrenal medulla [82] and decreasing 6-sulfatoxymelatonin, which is the primary metabolite of melatonin [83]. CAF disrupts the circadian rhythm by delaying the nighttime peak secretion of melatonin, which promotes the onset of these stages and causes difficulties getting to sleep as well as disruptions to sleep cycles [84,85]. 

While CAF seems to boost various aspects of athletic performance among female athletes, the observed side effects, such as nervousness and anxiety, are particularly notable when a moderate dosage of CAF is administered in the evening. These side effects could lead to a decrease in accuracy performance, which could significantly impact the technical and tactical aspects of ball game performance [3]. Thus, while the existing guidelines for athletes mainly discourage the consumption of high doses of CAF (≥9 mg/kg) [86] or stimulants near bedtime [87], the findings from this study underscore the necessity for more tailored recommendations regarding CAF intake. To develop practical guidelines for athletes, future research should examine the effects of CAF on sleep measurements at different doses, as well as the time of day, bedtime, and chronotype. Since our study supports the idea that while CAF supplements could improve physical performance, maintaining a careful balance is crucial to avoid potential adverse effects, and it is advisable to carefully schedule the administration of CAF during successive competitions or events.

### 4.5. Limitations

Although this pilot study, which involved female athletes, could open new avenues for further research on female team sports athletes, the present investigation has several limitations as follows: (a) because no biochemical analysis was assessed during the investigation, it was uncertain how differential doses affect variable CAF serum concentrations and women’s hormones across TOD; (b) the investigation did not include an assessment how the menstrual cycle affected performance or CAF effectiveness; (c) it is crucial to point out that because of different levels of fitness and because male athletes differ physiologically from female subjects due to innate physiological variations, the conclusions gained from our group of healthy, athletic women might not apply to non-athlete or male populations or ultra-endurance competitions overnight; and (d) it would be advantageous to evaluate the quality of sleep using objective measurements, such as actimetry or polysomnography. This would help to elucidate the effects of CAF intake across different times of day on sleep parameters. Unfortunately, due to logistical and technical constraints, this was not achievable. (e) Since our study included only low-CAF consumers, female athletes categorized as naive, moderate, or habitual CAF consumers may exhibit different responses to CAF. Therefore, further studies involving multiple female CAF consumers are necessary, as recently proposed [35]. Lastly, (f) the findings gathered from our participants may not be generalized to a larger population of female athletes displaying extreme chronotypes, using a contraceptive strategy or stimulants, or adhering to a rigorous food regimen. 

## 5. Conclusions

Although a moderate dose of 6 mg/kg of CAF significantly improved CMJ, agility, and repeated sprint performance compared to 3 mg/kg in the morning, with values close to those of the evening, neither dose effectively enhanced any of these performances in the evening. Neither CAF dose reduced the perceived effort scores or affected body temperature across various times of day. Additionally, afternoon CAF intake was linked to a greater occurrence of side effects, particularly after ingesting 6 mg/kg CAF. Thus, the current study supports the use of a moderate dose (6 mg/kg) of CAF consumption only during morning training or competition as an effective strategy to provide an overall ergogenic effect on short-term maximal performance among low-consumer female athletes of neither chronotype without causing severe negative side effects. This may be beneficial for athletes and coaches regarding the integration of CAF supplementation strategies as an ergogenic enhancer during congested training and tournaments. Athletes and coaches should require a strategic supplementation approach that considers the TOD of supplementation, the dose, and the time before habitual bedtime.

## Figures and Tables

**Table 1:** Side effects reported by the athletes immediately after the conclusion of each physical test battery (Q-D1) and 24 h later (Q-D2). The data are presented as percentages of prevalence (%). CAF: caffeine; D1: the same day; D2: the following day; mg: milligram; PLAC: placebo; CAF (3 mg): 3 mg/kg of CAF; CAF (6 mg): 6 mg/kg of CAF.

	PLAC				CAF (3 mg)				CAF (6 mg)			
	8:00 a.m.		6:00 p.m.		8:00 a.m.		6:00 p.m.		8:00 a.m.		6:00 p.m.	
Questionnaire	D1	D2	D1	D2	D1	D2	D1	D2	D1	D2	D1	D2
Muscle soreness	0	6.66	6.66	6.66	0	0	6.66	6.66	6.66	0	6.66	13.33
Increased urine output	0	6.66	0	6.66	0	13.33	13.33	20	6.66	6.66	6.66	26.66
Tachycardia	0	6.66	13.33	6.6	13.33	6.66	13.33	20	13.33	6.66	33.33	20
Anxiety or nervousness	6.66	6.66	0	6.66	0	6.66	6.66	6.66	6.66	0	13.33	13.33
Headache	6.66	6.66	6.66	0	13.33	6.66	13.33	26.66	20	13.33	20	40
Gastrointestinal problems	0	6.66	0	13.33	6.66	0	6.66	26.66	13.33	0	26.66	26.66
Insomnia	-	6.66	-	6.66	-	6.66	-	40	-	20	-	46.66
Increased vigor/activeness	6.66	0	13.33	0	6.66	6.66	13.33	6.66	13.33	6.66	13.33	13.33
Perception of performance improvement	13.33	-	6.66	-	13.33	-	13.33	-	13.33	-	13.33	-
Blinding	13.33		13.33		6.66		13.33		0		13.33	

## Data Availability

Data are available upon reasonable request from the first author. The data are not publicly available due to privacy reasons.
